# Long non-coding RNA GAS5 inhibits DDP-resistance and tumor progression of epithelial ovarian cancer via GAS5-E2F4-PARP1-MAPK axis

**DOI:** 10.1186/s13046-019-1329-2

**Published:** 2019-08-07

**Authors:** Xiaoran Long, Keqi Song, Hao Hu, Qi Tian, Wenjing Wang, Qian Dong, Xia Yin, Wen Di

**Affiliations:** 1 Department of Obstetrics and Gynecology, Ren Ji Hospital, School of Medicine, Shanghai Jiao Tong University, Shanghai, China; 2 Shanghai Key Laboratory of Gynecologic Oncology, Ren Ji Hospital, School of Medicine, Shanghai Jiao Tong University, Shanghai, China; 3 State Key Laboratory of Oncogenes and Related Genes, Shanghai Cancer Institute, Ren Ji Hospital, School of Medicine, Shanghai Jiao Tong University, NO.160, PuJian Road, Shanghai, China; 4 Department of Cancer Intervention, Ren Ji Hospital, School of Medicine, Shanghai Jiao Tong University, Shanghai, China

**Keywords:** lncRNA GAS5, PARP1, E2F4, MAPK, Ovarian cancer

## Abstract

**Background:**

Epithelial ovarian cancer (EOC) is the malignant tumor of the female reproductive system with the highest fatality rate. Tolerance of chemotherapeutic drugs like cisplatin (DDP) occurring in very early stage is one of the important factors of the poor prognosis of epithelial ovarian cancer. Here we aim to study the dysregulation of a particular long noncoding RNA, lncRNA GAS5, and its role in EOC progression.

**Methods:**

The low expression of lncRNA GAS5 in EOC tissues and OC cell lines was determined by microarray analyses and Real-Time qPCR. Flow cytometer assays were used to detect cell cycle and apoptosis of OC cells. CCK8 assay were performed to investigate the DDP sensitivity of OC cells. Western blot was carried out to detect cell growth markers, apoptotic markers, PARP1, E2F4, MAPK pathway protein expression and other protein expression in OC cell lines. The binding of GAS5 and E2F4 were proved by RNA pull-down and RIP assay. The effect of E2F4 on PARP1 were determined by CHIP-qPCR assay and luciferase reporter assay. The effect of lncRNA GAS5 on OC cells was assessed in vitro and in vivo.

**Results:**

By microarray (3 EOC tissues νs. 3 normal ovary tissues) and RT- qPCR (53 EOC tissues νs. 10 normal ovary tissues) we identified lncRNA GAS5 to be dramatically low expressed in EOC samples and correlated with prognosis. Compared with sensitive cell lines, GAS5 was also low expressed in DDP resistant OC cell lines, and over-expression of GAS5 significantly enhanced the sensitivity of OC cells to DDP in vivo and in vitro. Meanwhile the over-expression of GAS5 also caused OC cells G0/G1 arrest and apoptosis increase. Mechanistically, GAS5 might regulate PARP1 expression by recruiting the transcription factor E2F4 to its promoter, and then affect the MAPK pathway activity. Due to the 5’TOP structure, GAS5 could be regulated by transcription inhibitor rapamycin in OC cells.

**Conclusion:**

Here we explored the specific mechanisms of EOC cisplatin resistance and tumor progress due to lncRNA-GAS5, presented the GAS5-E2F4-PARP1-MAPK axis and its role in OC drug-sensitivity and progression for the first time, and the results may provide experimental basis for clinical application.

## Background

Ovarian cancer (OC) is considered to be the lethal gynecological cancer with the highest mortality rate worldwide, 5-year survival rate maintains at 25–30% [1, 2]. Epithelial ovarian cancer (EOC) accounts for 90% of OC cases in Asian populations [3]. In clinical practice, early metastasis and drug resistance result in poor prognosis of EOC [4]. At present, the clinical strategy for EOC is still cytoreductive surgery supplemented by platinum-based chemotherapy, while statistical studies have shown that about 15 to 25% of EOC patients appeared primary resistant to platinum-based chemotherapy, and at least 80% of all patients eventually develop secondary resistance to platinum [5]. Therefore, exploring the mechanism and regulation pathway of EOC drug resistance is essential to improve the survival benefit of EOC patients. In addition to the reduction of intracellular drug accumulation, increased drug efflux and drug inactivation caused by multi-drug resistance-related genes and their protein products such as P-glycoprotein (MDR1/P-gp), lung resistance associated protein (LRP), glutathione transferase (GST), etc.; and the classical resistance mechanisms such as DNA damage response pathway (DDR) activation; drug targets abnormalities or drug failure caused by abnormal regulation of apoptosis, metastasis or proliferation-related pathways are also new directions of recent researches on molecular mechanisms of EOC drug resistance. The epigenetic regulation of key genes and proteins on the above drug resistance related pathways has also become a hot spot in the study of tumor resistance interventions.

With the wide regulatory range, diverse regulatory pathways and specific targets, non-coding RNA (non-coding RNA) has become one important part of epigenetic researches. The aberrant expression of long non-coding RNA (lncRNA), a generic term for non-coding RNAs over 200 nucleotides in length, is involved in many disease processes [6], and is closely related to tumorigenesis, tumor progression and drug resistance [7–10]. In this study, we identified growth inhibition associated lncRNA GAS5 to be dramatically lower expressed in EOC and correlated with poor prognosis. LncRNA GAS5 is transcribed from the non-protein-coding snoRNA host gene GAS5, which is also known as the growth arrest-specific transcript 5 [11]. As a tumor suppressor-like lncRNA, its abnormal low expression has been found in various tumors including lung cancer [12], liver cancer [13], breast cancer [14], cervical cancer [15], etc. and participates in affecting tumor development process, immune regulation [16] and drug resistance [14]. Recent years, lncRNA-GAS5 has been reported to influence the cisplatin sensitivity in non-small cell lung cancer [17] and cervical cancer [18], and affected the adriamycin sensitivity in bladder transitional cell carcinoma as well [19]. But its influence and related mechanisms on progression or drug resistence in ovarian cancer are still unclear.

In this study, we confirmed the low-expression of lncRNA GAS5 in EOC tissues and OC cell lines, ascertained its tumor suppressor gene like role and discovered its sensitization function of cisplatin in ovarian cancer. And for the first time, we presented that GAS5 may regulate PARP1 expression by recruiting the transcription factor E2F4 to its promoter, and then affect MAPK pathway activity as well, thereby it may enhanced chemosensitivity by promoting apoptosis and causing cell cycle arrest of OC cells. In addition, because of its 5’TOP structure, the expression of GAS5 in cytoplasm could be elevated by translation inhibitor Rapamycin, and this intervention may be used to offset the low expression of GAS5 in ovarian cancer.

## Methods

### Cell culture

The human ovarian cancer cell lines HEY, A2780, A2780/DDP, HO8910, HO8910PM, SKOV3, SKOV3/DDP, and normal human ovarian epithelial cell line IOSE were maintained by our laboratory. Cells were cultured in RPMI− 1640 medium (Gibco, Grand Island, NY, USA), supplemented with 10% FBS (HyClone, Logan, UT, USA). All cells were cultured in a humidified atmosphere, at 37 °C with 5% CO_2_.

### Patients and specimens

Fifty three EOC tissues and 10 normal ovarian tissues were obtained from surgical specimens at Department of Obstetrics and Gynecology, Ren Ji hospital, School of Medicine, Shanghai Jiao Tong University (Shanghai, China) after informed consent during January 2013 to December 2015. All the specimens were staged according to FIGO classification of malignant Tumours-8th edition, and graded according to the 2018 world health

The inclusion and exclusion criteria of participants are as followed:

#### EOC group

**Inclusion criteria:**

**Pathology criteria:** Epithelial ovarian cancer, confirmed by two pathologists.

**Staging criteria:** I-IV (FIGO,2018).

**Surgical method:** Radical resection of ovarian cancer.

**Medical history:**

- ① No treatment with radiotherapy or chemotherapy before surgery.
- ② No death occurring within 1 month after surgery.
- ③ Clinicopathological characteristics and follow-up information available.

**Requirements for the tissue samples:**

- ① Derived from the first surgery.
- ② RNA concentration more than 200 ng/μl.

**Exclusion criteria:**

- ① Death within 1 month after operation.
- ② Lost cases.
- ③ RNA quality is not up to standard.

#### Normal group

**Inclusion criteria:**

**Pathology criteria:** Normal ovarian cancer, confirmed by two pathologists.

**Surgical method:** Gynecological malignancies or benign diseases except ovarian cancer undergoing total hysterectomy and adnexal resection.

**Requirements for the tissue samples:**

- ① Derived from the first surgery.
- ② RNA concentration more than 200 ng/μl.

**Exclusion criteria:**

- ① Lesion invade ovaries.
- ② RNA quality is not up to standard.

#### Sample collection

All samples derived from surgery were cut into two parts, each of which was no less than 0.5 cm*0.5 cm*0.5 cm. One part was immediately placed in a test tube containing RNA later for RNA extraction, and the other part is immediately stored in an empty test tube in a − 80 °C refrigerator. Cutting and transferring were done on ice.

The Institutional Review Board (IRB) approval number of this work is [2018]114 (Issuance of Ethics Approval by Ethics Committee of Renji Hospital).

### Microarray analysis

LncRNAs in the RNA samples were profiled using human SBC Human (4*180 K) ceRNA microarray analysis (Bio-tech, Shanghai, China). Quantile normalization and data processing were performed using the GeneSpring GX v11.5.1 software package (Agilent Technologies). Differentially expressed lncRNAs were identified by Heatmap. Up-regulated or down-regulated lncRNAs were selected based on changes ≥2 fold threshold and *P* < 0.05.

### Real-time reverse transcription polymerase chain reaction

The relative quantity of lncRNA and mRNA were measured by RT-qPCR. The gene-specific primers were designed by Primer Premier 5.0 software (Premier Biosoft International, Palo Alto,CA, USA).

### Western blot

Total protein was separated by 10% SDS-PAGE and transferred into polyvinylidene fluoride membranes (Millipore, Billerica, MA, USA). After that, the membranes were blocked by 5% nonfat milk, and incubated with primary antibodies (anti-PARP1, anti-E2F4, anti-CDK4, anti-CDK6, anti-CyclinD, anti-CASP3, anti-cleaved CASP3, anti-CASP7, anti-cleaved CASP7, anti-GAPDH, anti-β-actin [Abcam, USA], anti-p-ERK, anti-p-ERK, anti-p-JNK and anti-P38MAPK [Cell Signaling Technology, USA]), at 4 °C overnight. The membranes were then incubated with anti-rabbit/mouse IgG secondary antibody (CST, USA). The protein bands were visualized using enhanced chemiluminescent substrate (ECL, Pierce, USA).

### Flow cytometry

Cell apoptosis was evaluated using the Annexin-V/Propidium Iodide Detection Kit (Key GEN, China). Cells were analyzed by FACS cytometry (BD Biosciences Inc.)

### Luciferase reporter assay

The PARP1 sequence containing predicted E2F4 binding site was sub-cloned and inserted into pmirGLO vector (Promega, Madison, WI, USA). After that, SKOV3/HEY cells were incubated in a 96-well plate and co-transfected with recombinant plasmids (50 nM) or empty pmirGLO vector (200 ng), After incubation for 48 h, the cells were lysed in 1x Passive lysis. The Dual-Luciferase Reporter Assay System (Promega) was applied to measure the Renilla luciferase activity, with firefly luciferase serving as a transfection control.

### RNA immunoprecipitation (RIP)

RIP experiments were performed using Magna RIP RNA-Binding Protein Immunoprecipitation Kit (Millipore, Billerica, MA, USA) according to the manufacturer’s instructions. The co-precipitated RNAs were detected by reverse transcription PCR. To demonstrate that the detected signals were from the RNA that was specifically bound, total RNA (input controls) and corresponding species IgG controls were performed simultaneously (*n* = 3). For the anti-E2F4 RIP experiment, GAS5 were transiently transfected into SKOV3 and HEY cells. Cells were harvested and lysed for RIP using anti-E2F4 antibody after 48 h of transfection

### Chromatin immunoprecipitation (ChIP)

ChIP assays were performed using the EZ-ChIP chromatin immunoprecipitation kit (Millipore, Billerica, MA, USA) according to the manufacturer’s guidelines. Immunoselections of cross-linked protein-DNA were performed using anti-PARP1 antibody together agarose beads A/G, at 4 °C for overnight. The anti-rabbit IgG was used as a negative control. The purified DNAs were analyzed by PCR, the forward and reverse primers were: 5′- CTGATGTTGCAGGAAAAGCCC-3′ and 5′- AATAAAACACCGCCACCCAGA-3′ for human PARP1 promoter.

### RNA pull-down assay

Cellular nuclear protein was extracted by using the NE-PER Nuclear and Cytoplasmic Extraction Reagents (Thermo Fisher Scientific, Inc., USA), and then incubated with biotin-labeled GAS5 truncation probe and streptavidin agarose beads (Invitrogen). Finally, the retrieved protein was detected by Western-blot.

### Tumorigenicity assays in nude mice

All animal studies were conducted in accordance with institutional guidelines for animal care and were approved by the committee for the use and care of animals of Renji hospital (Shanghai, China). The Institutional Review Board (IRB) approval number of this work is [2018]114 (Issuance of Ethics Approval by Ethics Committee of Renji Hospital). Briefly, SKOV3 cells were first transfected with the GAS5 expression vector or with empty vector for 72 h. The cells were then harvested at the exponential growth stage when they reached 90% confluence. Approximately 2.5 × 10^6^ cells in 50% Matrigel were injected subcutaneously into the right flanks of 4- to 6-week-old nude mice. Tumor growth was monitored, and tumor sizes were measured every other day using caliper. The tumor volume (V) was calculated using the formula: V = 1/2(length × width^2^).In the DDP treatment groups, 5 mg/kg DDP was injected through tail vein every 3 days. When the maximum tumor size ≥1000 mm^3^, end monitor and execute mice.

### Immunohistochemistry

Tissue samples were embedded in paraffin. Expression of CDK4, Ki67 and PARP1 were detected by immunohistochemical staining. Sections were visualized under a microscope (400× or 200×) (Olympus, Japan). The results were graded according to the percentage of positive cells.

### RNA-fish

Cy3-labeled GAS5 and DAPI-labeled U6 probes were obtained from RiboBio (Guangzhou, China). RNA FISH were performed using fluorescent in situ hybridization kit (RiboBio) according to manufacturer’s instructions.

### Statistical analysis

Statistical analysis was performed using the statistical program SPSS 13.0. Quantitative results were expressed as the mean ± standard error of mean (SEM) and were analyzed using Student’s t test. *p* value < 0.05 were considered statistically significant.

## Results

### LncRNA GAS5 was down-regulated in EOC tissues and OC cell lines, and associated with EOC prognosis

**Fig. 1:**
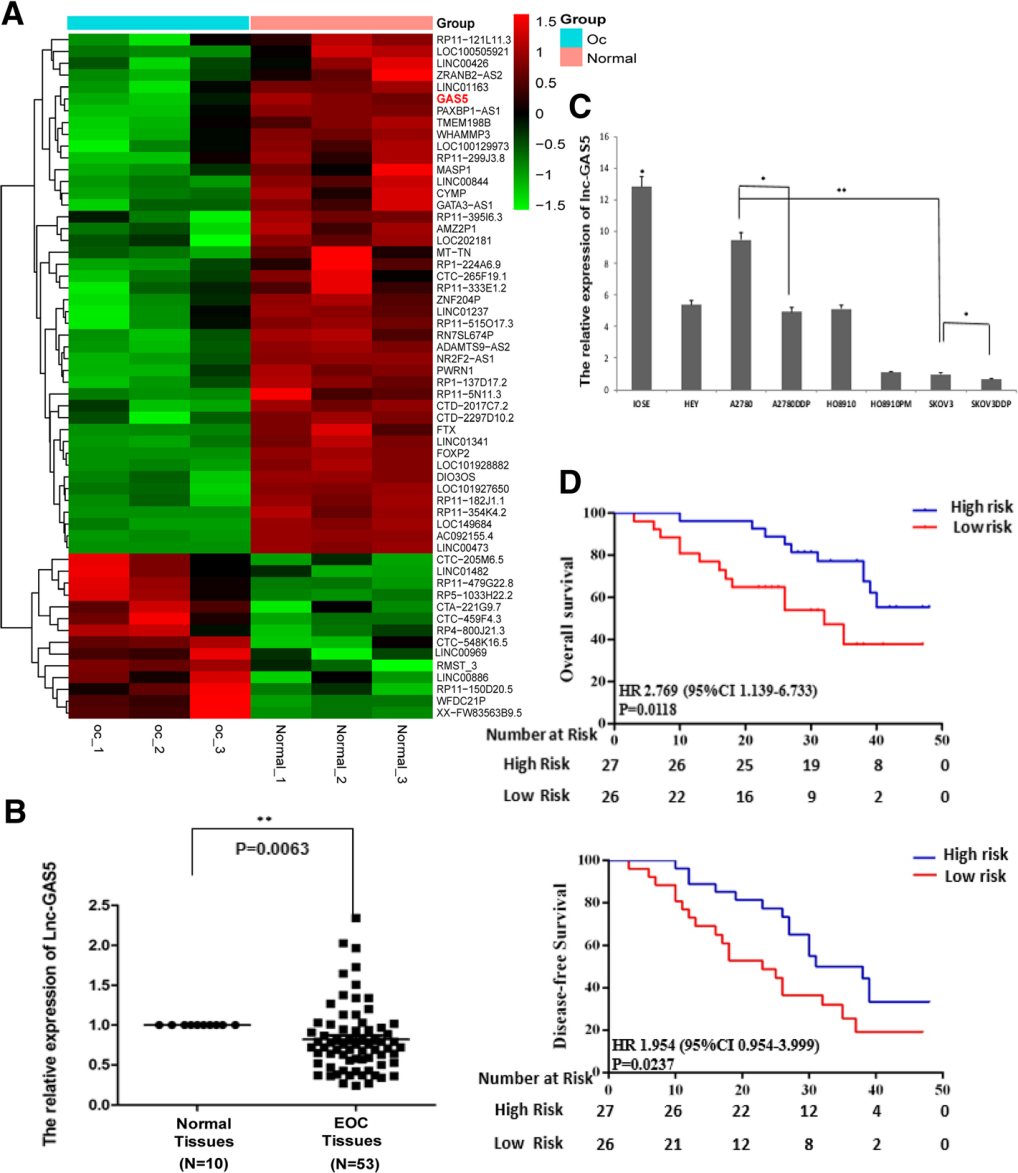
LncRNA GAS5 was down-regulated in EOC tissues and OC cell lines, and associated with EOC prognosis. **a** Hierarchical clustering of Agilent lncRNA array result showing abnormal expressed lncRNAs in normal ovarian or EOC tumor tissues. GAS5 is one of the most low-expressed lncRNAs in EOC tissues. **b** Total RNA was extracted from human normal ovarian tissues (Normal) of 10 participants and EOC tumor tissues (OC Tissues) of 53 patients, GAS5 expression levels were then detected by RT-qPCR assay. **c** GAS5 expression levels in OC cell lines and normal human ovarian epithelial cell line were detected by RT-qPCR assay. **d** Kaplan–Meier overall survival curves (OS) and disease-free survival curves (DFS) was observed based on GAS5 level. Data were the means±SD of triplicate determinants. *P < 0.05 versus control groups. **P < 0.005 versus control groups

Microarray (3 EOC tissues νs. 3 normal ovary tissues) results determined that the expression of lncRNA GAS5 was significantly decreased in EOC tissues compared with normal ovarian tissues (OC νs. Normal fold change = 0.003, *p* value = 0.028) (Fig. 1a). RT-qPCR also showed that compared with 10 normal ovarian tissues, GAS5 was remarkably low-expressed in 53 EOC tissues (*p* value = 0.006) (Fig. 1b). And the expression level of GAS5 in seven OC cell lines (HEY, A2780, A2780/DDP, HO8910, HO8910PM, SKOV3, SKOV3/DDP) were also dramatically lower than that in normal human ovarian epithelial cell line IOSE (Fig. 1c). Furthermore, Kaplan-Meier survival analysis revealed that the low expression of GAS5 was associated with low overall survival rate (p value = 0.012) and disease-free survival rate (p value = 0.024) of OC patients (Fig. 1d), and as shown in Table 1, as an independent prognostic factor, GAS5 expression was significantly correlated with advanced FIGO stage and histological type. This result suggested that GAS5 downregulation might be vital in the progression of ovarian cancer.

**Table 1:** Clinical characteristics of EOC patients with high and low GAS5 risk scores

	Case(n)	GAS5		P
		Low-risk (n)	High-risk (n)	
Age				
≥ mean	33	17	16	0.269
< mean	20	8	12	
FIGO Stage				
I- II	22	16	6	0.007*
III-IV	31	10	21	
Histological Type				
I	11	7	4	0.024*
II- III	42	16	26	
Residual tumor (cm)				
< 1 cm	34	18	16	0.332
≥ 1 cm	19	8	11	
Histology				
Mucinous	7	3	4	0.402
Serous	41	17	24	
Endometrioid	3	1	2	
Clear cell	2	1	1	
Lymphatic metastasis				
No	20	8	12	0.257
Yes	33	14	19	
Ascites				
No	21	14	7	0.096
Yes	32	13	19	

*P* Values are calculated by X^2^ test or Fisher’s exact test

### GAS5 over-expression caused cell cycle arrest and promoted apoptosis of OC cells

**Fig. 2:**
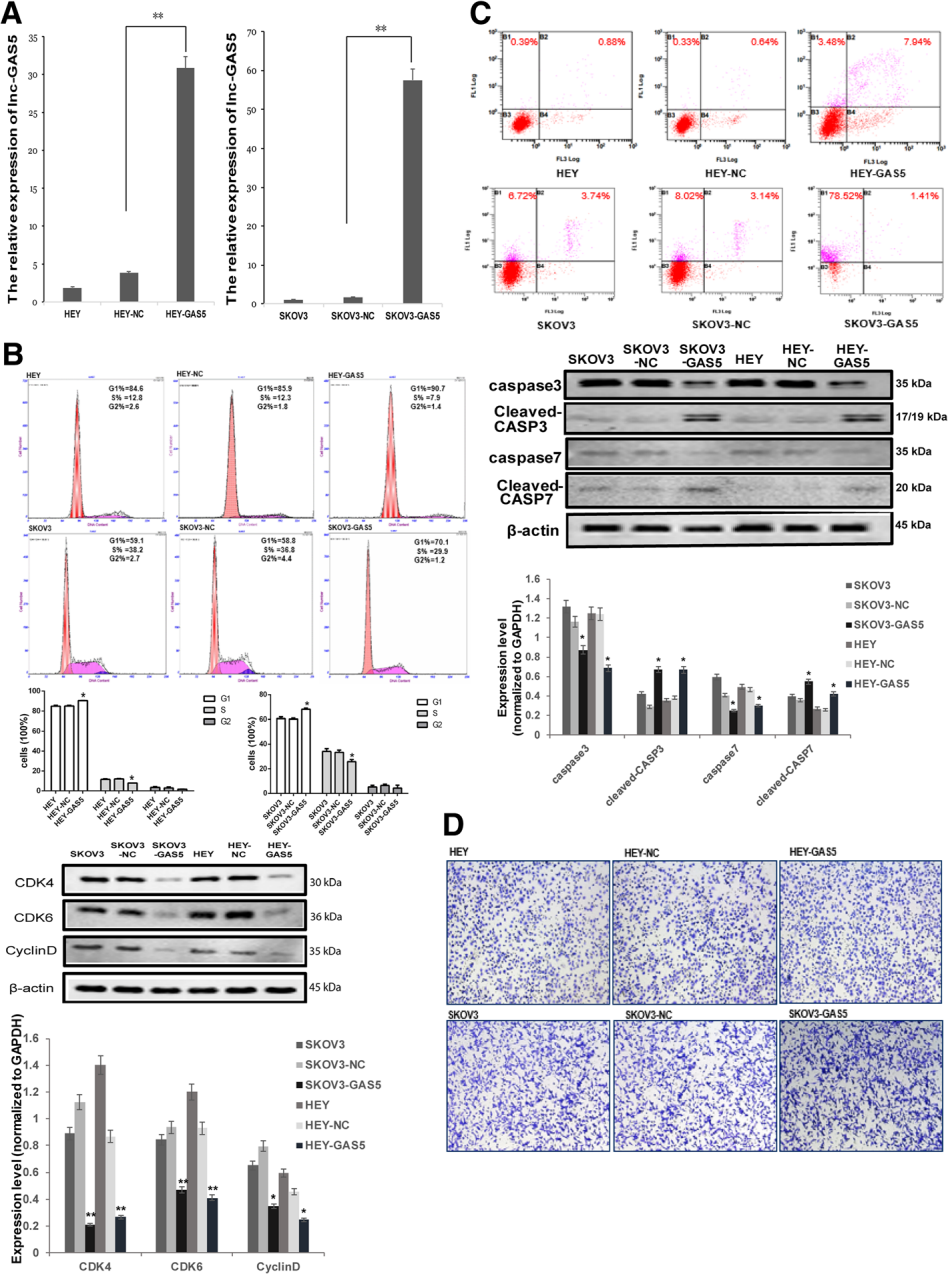
GAS5 over-expression caused cell cycle arrest and promoted apoptosis of OC cells. **a** LV5-GAS5 or the empty LV5 vector plasmids were transfected into OC cells. 72 h later, overexpression efficient of GAS5 was evaluated by RT-qPCR. **b** 72 h after transfection of LV5-GAS5 or the empty vector, flow cytometry assay were performed to detect the cell cycle impacted by overexpression of GAS5 in OC cell lines SKOV3 and HEY, and cell cycle-related protein levels were then assessed by Western-blot assay. β-actin was served as the internal control. **c** After transfection, cells were harvested and stained by Annexin V-PE and 7-AAD, and apoptosis rates were analyzed by flow cytometry, and apoptosis-related protein levels were then assessed by Western-blot assay. β-actin was served as the internal control. **d** Transwell assay were performed to detect the migration rate of SKOV3 and HEY. *P < 0.05 versus control groups

After constructed a retroviral stable expression of GAS5 in HEY and SKOV3 cells, qRT-PCR were performed to validate the over-expression levels of GAS5 in these two cell lines (Fig. 2a). As shown by the results of the flow cytometry, over-expression of GAS5 caused G0/G1 cell cycle arrest and apoptosis increase. In the meantime, Western-blot showed that GAS5 overexpression affects the expression of cell cycle and apoptosis-related proteins (Fig. 2b and c). However, overexpression of GAS5 had little impact on OC cells migration rate (Fig. 2d). These results indicated that GAS5 worked as a tumor suppressor gene, and played a specific role in regulating the cell cycle and apoptosis but not the migration of OC cells in vitro.

### GAS5 over-expression enhanced sensitivity to DDP of OC both in vitro and in vivo

**Fig. 3:**
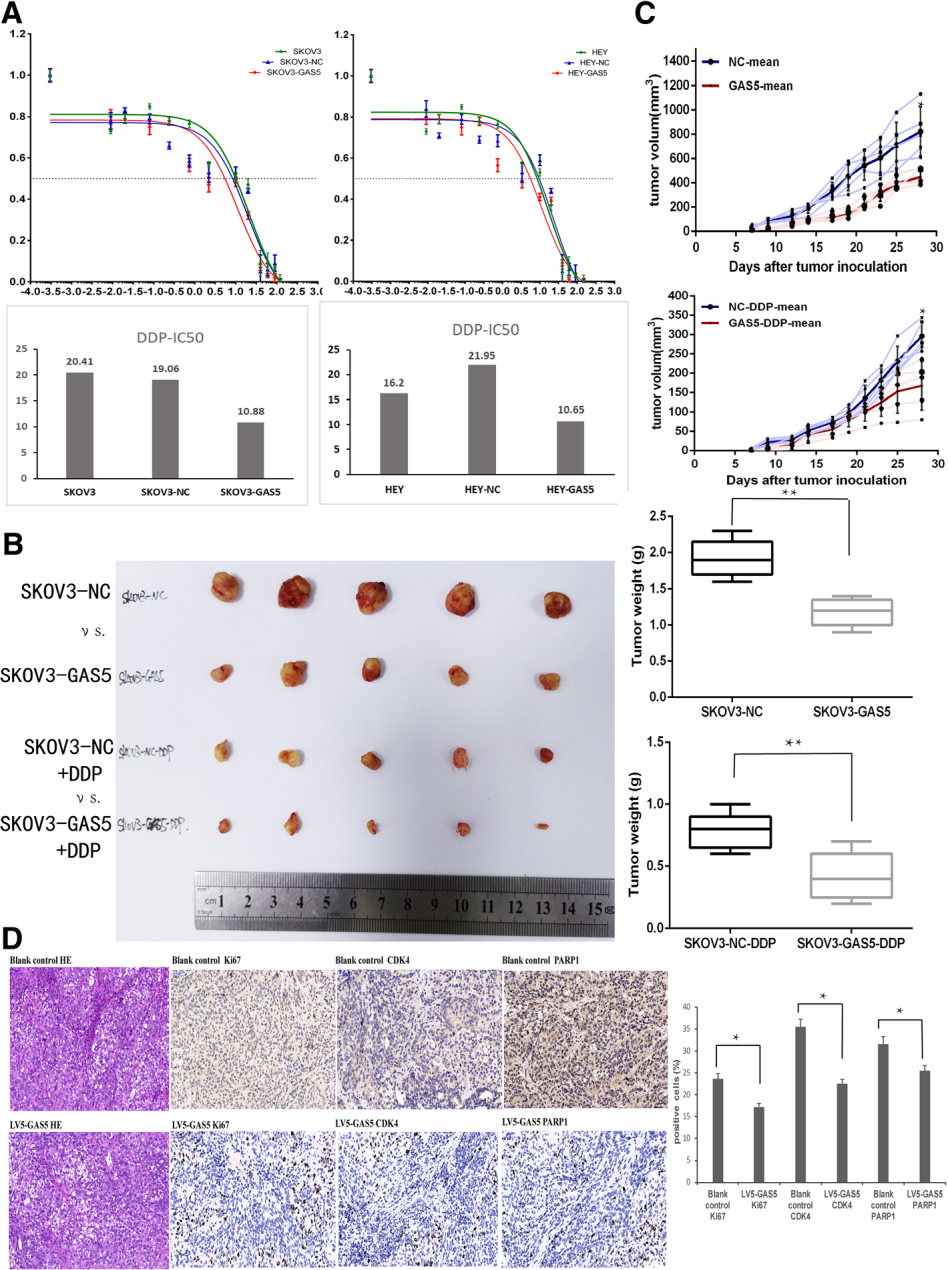
GAS5 over-expression enhanced sensitivity to DDP in OC cells both in vitro and in vivo. **a** DDP sensitivity were detected by CCK8 assay and IC50 in OC cell lines SKOV3 and HEY. **b** The tumor tissues of nude mice were presented. **c** The volume of tumor was calculated on the 7, 9, 12, 14, 17, 19, 21, 23, 25 and 28 days. And the tumor weight were measured when tumors were harvested. **d** Immunohistochemical staining were performed to detect the Ki67, CDK4 and PARP1 expression in animal tumor tissues. *P < 0.05 versus control groups

As shown in the qRT-PCR assay, GAS5 expression in DDP-resistant OC cell lines was significantly lower than in DDP-sensitive cells (A2780DDP vs. A2780, SKOV3DDP vs. SKOV3, A2780 vs. SKOV3) (Fig.1c). We then meditated that GAS5 might be correlated with DDP resistant in OC. As expected, HEY and SKOV3 cells stably expressing GAS5 showed to be more sensitive to DDP compared with control group in vitro (Fig.3a). When injected into nude mice, the tumor size and weight of stably expressing GAS5 groups were all remarkably lessened than the control groups 4 weeks after injection, and GAS5 overexpression groups seems more sensitive to DDP compared with control group (Fig. 3b and c). Furthermore, the immunohistochemical staining of Ki67, CDK4 and PARP1 showed that the proportion of positive cells of these protein in GAS5 over-expression group were lower than those in NC group (Fig.3d). Taken together, our data showed that GAS5 suppressed tumor growth and enhanced the sensitivity of cisplatin (DDP) in ovarian cancer.

### GAS5 regulated the expression of PARP1 and effected MAPK pathway in OC cells

**Fig. 4:**
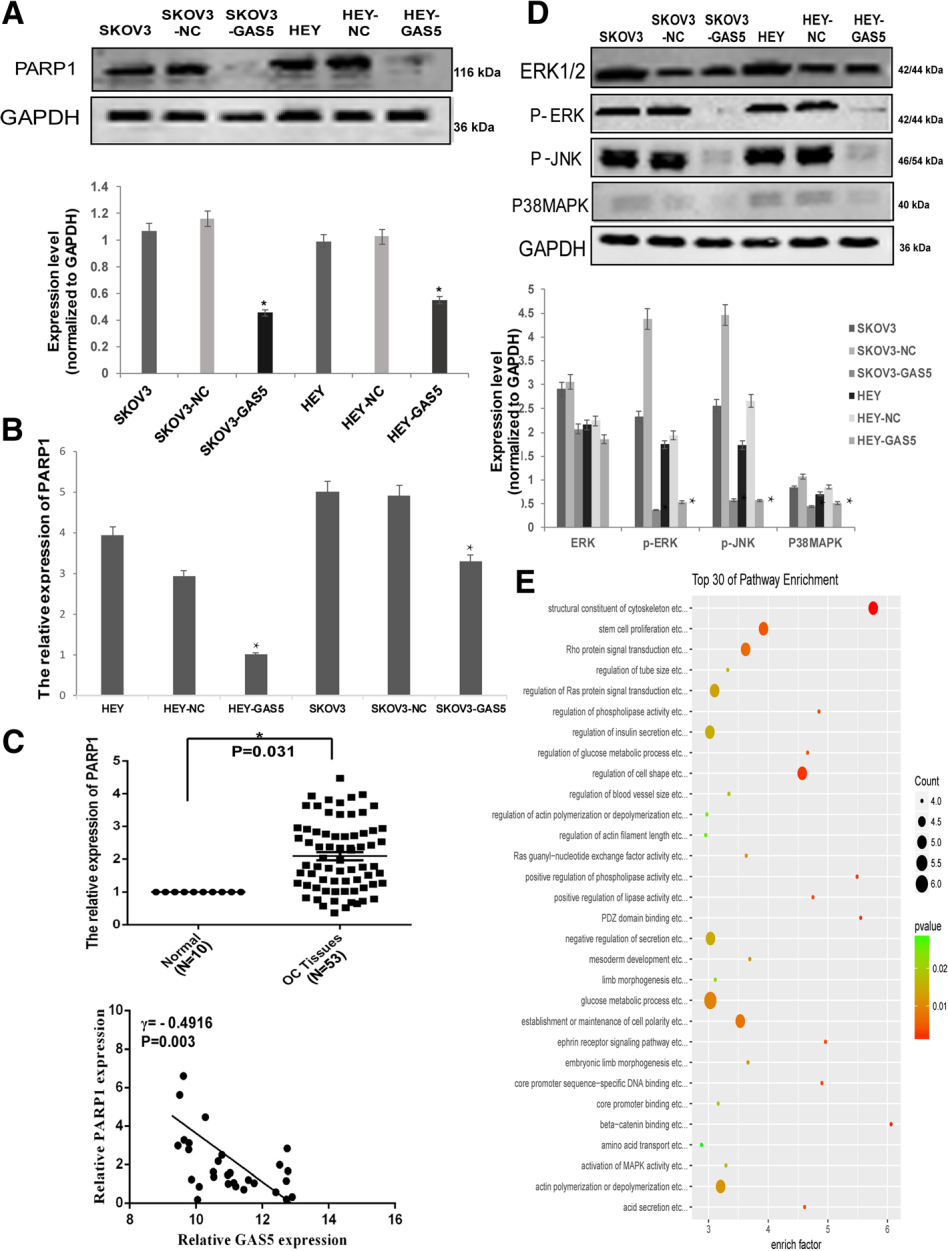
GAS5 regulated the expression of PARP1 and effected MAPK pathway in OC cells. **a** The protein level of PARP1 were assessed by Western-blot assay. GAPDH was served as the internal control. **b** The mRNA level of PARP1 were assessed by RT-qPCR assay. **c** PARP1 transcriptional activities of OC cells stably transfected with LV5-GAS5 or the empty vector were assessed by luciferase reporter assay. **d** The protein level of MAPK pathway members were assessed by Western-blot assay. GAPDH was served as the internal control. **e** GO analysis showed the top 30 pathway enrichment affected by GAS5 over-expression. *P < 0.05 versus control groups

Poly (ADP-ribose) Polymerase1 (PARP1) promotes tumor progress via diversified biological functions such as cell cycle regulation, DNA repair, apoptosis inhibition, and maintaining MAPK activity, etc. [20] Clinically, PARP1 inhibitors have shown their potential in treating breast and ovarian cancers [21]. In this study, the clustering analysis of whole genome microarray data predicted that GAS5 may affect PARP1 Bind Protein (PARPBP), we then found that forced expression of GAS5 markedly down-regulated the protein and mRNA level of PARP1 in OC cells (Fig. 4a and b). As shown in RT-qPCR assay, PARP1 was high expressed in 53 EOC patient samples (*P* = 0.031), negatively related with GAS5 expression level (γ = − 0.4916, *P* = 0.003) (Fig. 4c). PARP1 has been reported to be indispensible for maintaining MAPK activity. Western-blot showed that GAS5 overexpression indeed affected the phosphorylation level of MAPK pathway members (Fig. 4d), as assumed by GO analysis of microarray data (Fig. 4e).

### GAS5 regulated the PARP1 transcriptional by recruiting the transcription factor E2F4 to its promoter in OC cells

**Fig. 5:**
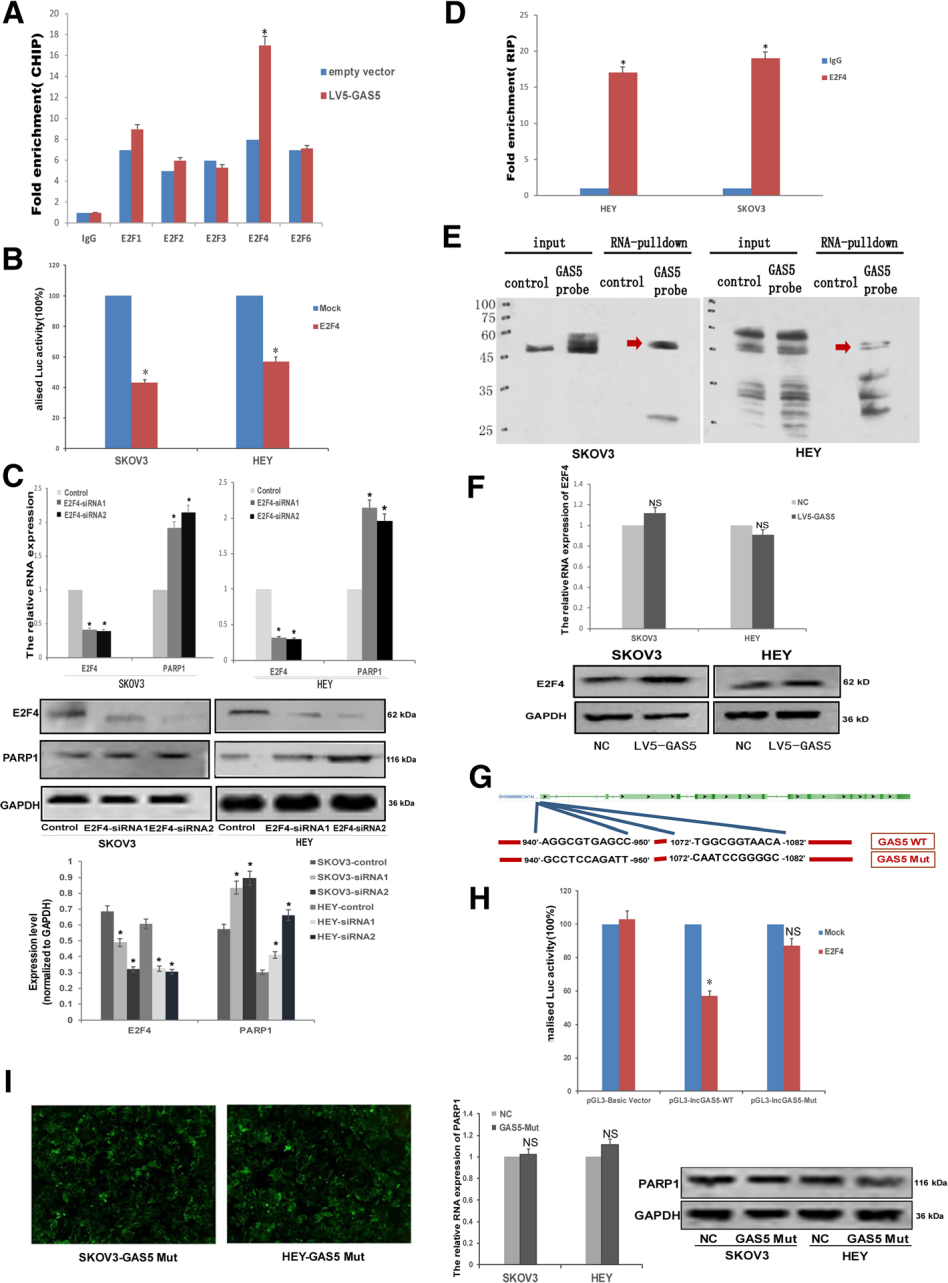
GAS5 affected the PARP1 transcriptional expression by recruiting the transcription factor E2F4 to its promoter in OC cells. **a** LV5-GAS5 or the empty vector was transfected into OC cells, and chrome immunoprecipitations (CHIP) were performed by using specific anti-E2F1, anti-E2F2, anti-E2F3, anti-E2F4 or anti-E2F6 antibodies. **b** PARP1 luciferase reporter plasmid was used for luciferase reporter assay, and co-transfected E2F4 resulted in a decreased promoter activity of PARP1 in HEY and SKOV3 cells. **c** E2F4 siRNAs (siE2F4–1, 2) or the control siRNA were transfected into OC cells for 36 h, E2F4 and PARP1 mRNA and protein levels were then assessed by RT-qPCR or Western-blot assay. GAPDH was served as the internal control. **d** RNA immunoprecipitations (RIP) were performed in OC cells, and the relative quantities of GAS5 were detected by RT-qPCR assay, normalized to the input groups. IgG and E2F4 represented for the groups coprecipitation with IgG protein and anti-E2F4 antibody respectively. **e** Total proteins were extracted from SKOV3 and HEY cells, and then lncRNA GAS5 pull-down assay was performed. The E2F4 protein levels were evaluated by Western-blot. GAS5 probe represented the biotin-labeled GAS5 probe group and control stood for the oligo probe group. **f** After forced over-expression of GAS5, the mRNA and protein level of E2F4 were assessed by RT-qPCR and Western-blot assay. **g** Sketch map of GAS5 mutated E2F4 binding sites. **h** luciferase reporter assay: mutation type GAS5 (GAS5 Mut) plasmid co-transfected with E2F4 manifested no changes in GAS5 promoter activity in 293 cells. **i** After stable transfect mutation type GAS5 (GAS5 Mut) plasmid, the mRNA and protein level of PARP1 were assessed by RT-qPCR and Western-blot assay in SKOV3 and HEY cell lines. *P < 0.05 versus control groups

While PARP1 transcription has been reported to be repressed by E2Fs complex in human monocytes [22], in hypoxic cancer cells [23], and in bladder cancer [24]. We found that there were 69 E2Fs-binding sites in PARP1 promoters by Jaspar matrix models (http://jaspar.genereg.net/) (Table 2). The E2F family is mainly comprised of 8 members (E2F1–8). Though both E2F1, E2F2, E2F3, E2F4 and E2F6 could bind to PARP1 promoter, the CHIP-qPCR assay showed that in OC cells, only E2F4’s binding capacity was improved evidently with GAS5 over-expression (Fig. 5a). And there are 13 E2F4-binding sites in PARP1 promoters predicted by Jaspar (Table 3). Therefore we focus E2F4 as the target of our study. To illustrate the mechanism of the regulation of PARP1 expression by E2F4, human PARP1 luciferase reporter plasmid was used for luciferase reporter assay, and co-transfected E2F4 resulted in a decreased promoter activity of PARP1 in HEY and SKOV3 cells (Fig. 5b). E2F4 was then knockdown by siRNA, and the results of RT-qPCR and western blot revealed that PARP1 expression was significantly upregulated (Fig. 5c). And then RNA immunoprecipitation (RIP) assay was performed, which demonstrated that E2F4 protein could bind to GAS5 (Fig. 5d) as well. The direct interaction between E2F4 and GAS5 was further verified by applying total protein to GAS5 pull-down assay (Fig. 5e). In addition, we found GAS5 over-expression did not impact the expression of E2F4 protein and mRNA (Fig. 5f). To explored whether the combination of GAS5 with E2F4 is essential for regulating PARP1 expression, we checked the possible binding sites for E2F4 in GAS5 promoter predicted by Jaspar (Table 4) and constructed mutation type (Mut) GAS5 retroviral plasmid and luciferase plasmid (Fig. 5g). As shown by luciferase reporter assay, mutation type GAS5 (GAS5 Mut) plasmid co-transfected with E2F4 manifested no changes in GAS5 promoter activity in 293 cells, indicated that the mutation of the binding sites abolish the combination of GAS5 with E2F4 (Fig. 5h). While stable transfect mutation type GAS5 (GAS5 Mut) plasmid in SKOV3 and HEY cell lines (Fig. 5i, left), RT-qPCT and Western blot assay indicated that mutation type GAS5 transfection lost the effect on PARP1 expression (Fig. 5i, middle and right). Taken together, we concluded that GAS5 inhibited PARP1 transcriptional activity by recruiting transcription factor E2F4 to its promoter.

**Table 2:** Possible binding sites of E2Fs in PARP1 promoter

Model ID	Model name	Score	Relative score	Start	End	Strand	Predicted site sequence
MA0024.2	E2F1	3.427	0.820	35	45	−1	TGAGCTGCAGC
MA0024.1	E2F1	8.832	0.870	86	93	1	TTTGGGGC
MA0024.2	E2F1	5.271	0.847	102	112	1	TGGGTGCCAGG
MA0471.1	E2F6	8.684	0.889	375	385	−1	AAGTGGGAGGA
MA0024.2	E2F1	4.624	0.838	376	386	−1	GAAGTGGGAGG
MA0470.1	E2F4	4.217	0.812	388	398	−1	ACTCGGGAGGC
MA0471.1	E2F6	4.490	0.827	388	398	−1	ACTCGGGAGGC
MA0024.2	E2F1	5.067	0.844	389	399	−1	TACTCGGGAGG
MA0471.1	E2F6	2.914	0.804	415	425	−1	TGGTGGCAGGT
MA0024.2	E2F1	5.276	0.847	416	426	−1	GTGGTGGCAGG
MA0469.1	E2F3	7.755	0.840	909	923	−1	CTCCCACCTCGACTT
MA0024.2	E2F1	6.718	0.869	914	924	1	GAGGTGGGAGG
MA0470.1	E2F4	5.788	0.836	915	925	1	AGGTGGGAGGA
MA0471.1	E2F6	10.763	0.920	915	925	1	AGGTGGGAGGA
MA0024.2	E2F1	6.352	0.864	1238	1248	1	TGGACGGCAGG
MA0471.1	E2F6	5.290	0.839	1269	1279	1	AGGAGGGTGGA
MA0024.2	E2F1	5.156	0.846	1299	1309	−1	TTGGCCCGAGG
MA0469.1	E2F3	5.736	0.813	1319	1333	− 1	CCCCCGCCTCGGGAA
MA0024.2	E2F1	5.886	0.857	1324	1334	1	GAGGCGGGGGC
MA0470.1	E2F4	7.463	0.863	1325	1335	1	AGGCGGGGGCC
MA0471.1	E2F6	5.390	0.841	1325	1335	1	AGGCGGGGGCC
MA0471.1	E2F6	6.073	0.851	1364	1374	1	GGGAGAGAGGA
PB0112.1	E2F2_2	7.546	0.807	1384	1400	−1	ACGCCGGCCCCAAACTC
MA0024.1	E2F1	8.832	0.870	1387	1394	1	TTTGGGGC
MA0469.1	E2F3	5.222	0.806	1434	1448	−1	TTCACGCCTCAGCCT
MA0024.2	E2F1	7.150	0.876	1439	1449	1	GAGGCGTGAAG
MA0470.1	E2F4	6.980	0.855	1440	1450	1	AGGCGTGAAGA
MA0471.1	E2F6	7.460	0.871	1440	1450	1	AGGCGTGAAGA
MA0024.2	E2F1	2.878	0.811	1487	1497	−1	GTCTCGCCAAG
MA0024.2	E2F1	5.067	0.844	1560	1570	1	TACTCGGGAGG
MA0470.1	E2F4	4.217	0.812	1561	1571	1	ACTCGGGAGGC
MA0471.1	E2F6	4.490	0.827	1561	1571	1	ACTCGGGAGGC
MA0469.1	E2F3	5.644	0.811	1568	1582	−1	CTCCCACCTCAGCCT
MA0024.2	E2F1	6.718	0.869	1573	1583	1	GAGGTGGGAGG
MA0470.1	E2F4	5.788	0.836	1574	1584	1	AGGTGGGAGGA
MA0471.1	E2F6	10.763	0.920	1574	1584	1	AGGTGGGAGGA
PB0112.1	E2F2_2	8.488	0.826	1619	1635	−1	GCAGTGCCGCCATCATG
PB0112.1	E2F2_2	7.730	0.810	1620	1636	1	ATGATGGCGGCACTGCA
PB0113.1	E2F3_2	7.920	0.808	1620	1636	1	ATGATGGCGGCACTGCA
MA0470.1	E2F4	3.684	0.803	1635	1645	−1	GCGCTGGAGTG
MA0471.1	E2F6	3.063	0.806	1635	1645	−1	GCGCTGGAGTG
MA0469.1	E2F3	5.667	0.812	1637	1651	1	CTCCAGCGCGGTGAG
MA0024.2	E2F1	4.515	0.836	1669	1679	1	AAAGGGGGAGG
MA0471.1	E2F6	5.461	0.842	1670	1680	1	AAGGGGGAGGG
PB0112.1	E2F2_2	9.187	0.840	1738	1754	−1	GACCCGGCGCCACCCCT
PB0113.1	E2F3_2	10.273	0.856	1738	1754	−1	GACCCGGCGCCACCCCT
MA0024.2	E2F1	4.195	0.831	1742	1752	1	GTGGCGCCGGG
MA0024.2	E2F1	4.281	0.832	1821	1831	1	CACCCGGCAGG
MA0024.2	E2F1	5.062	0.844	1827	1837	−1	CGGGCGCCTGC
MA0024.2	E2F1	4.402	0.834	1828	1838	1	CAGGCGCCCGG
MA0024.2	E2F1	6.411	0.864	1832	1842	1	CGCCCGGGAAA
MA0470.1	E2F4	7.805	0.868	1833	1843	1	GCCCGGGAAAC
MA0471.1	E2F6	5.723	0.845	1833	1843	1	GCCCGGGAAAC
MA0024.1	E2F1	7.993	0.841	1835	1842	−1	TTTCCCGG
MA0470.1	E2F4	3.661	0.803	1847	1857	−1	GGCCGGGGGGC
MA0024.2	E2F1	6.975	0.873	1853	1863	1	CGGCCGGCAGG
MA0470.1	E2F4	3.553	0.801	1854	1864	1	GGCCGGCAGGG
PB0008.1	E2F2_1	8.763	0.811	1860	1874	1	CAGGGGGCGCGCGCG
PB0009.1	E2F3_1	9.191	0.826	1860	1874	1	CAGGGGGCGCGCGCG
MA0024.2	E2F1	6.297	0.863	1863	1873	1	GGGGCGCGCGC
PB0008.1	E2F2_1	8.245	0.800	1863	1877	−1	CGGCGCGCGCGCCCC
MA0470.1	E2F4	3.869	0.806	1864	1874	1	GGGCGCGCGCG
MA0024.2	E2F1	5.095	0.845	1865	1875	1	GGCGCGCGCGC
MA0024.2	E2F1	5.095	0.845	1866	1876	−1	GGCGCGCGCGC
MA0470.1	E2F4	3.825	0.805	1879	1889	−1	GGGCGGGGCCG
MA0470.1	E2F4	12.687	0.946	1911	1921	−1	CCGCGGGAACG
MA0471.1	E2F6	8.773	0.890	1911	1921	−1	CCGCGGGAACG
MA0024.2	E2F1	6.807	0.870	1912	1922	-1	GCCGCGGGAAC
MA0469.1	E2F3	13.658	0.920	1913	1927	1	TTCCCGCGGCCAGGC

69 putative sites were predicted with these settings (80%) in sequence named NC_000001.11:c226408100–226360691

**Table 3:** Possible binding sites of E2F4 in PARP1 promoter

Model ID	Model name	Score	Relative score	Start	End	Strand	Predicted site sequence
MA0470.1	E2F4	4.217	0.812	388	398	-1	ACTCGGGAGGC
MA0470.1	E2F4	5.788	0.836	915	925	1	AGGTGGGAGGA
MA0470.1	E2F4	7.463	0.863	1325	1335	1	AGGCGGGGGCC
MA0470.1	E2F4	6.980	0.855	1440	1450	1	AGGCGTGAAGA
MA0470.1	E2F4	4.217	0.812	1561	1571	1	ACTCGGGAGGC
MA0470.1	E2F4	5.788	0.836	1574	1584	1	AGGTGGGAGGA
MA0470.1	E2F4	3.684	0.803	1635	1645	-1	GCGCTGGAGTG
MA0470.1	E2F4	7.805	0.868	1833	1843	1	GCCCGGGAAAC
MA0470.1	E2F4	3.661	0.803	1847	1857	-1	GGCCGGGGGGC
MA0470.1	E2F4	3.553	0.801	1854	1864	1	GGCCGGCAGGG
MA0470.1	E2F4	3.869	0.806	1864	1874	1	GGGCGCGCGCG
MA0470.1	E2F4	3.825	0.805	1879	1889	-1	GGGCGGGGCCG
MA0470.1	E2F4	12.687	0.946	1911	1921	-1	CCGCGGGAACG

13 putative sites were predicted with these settings (80%) in sequence named NC_000001.11:c226408100–226360691

**Table 4:** Possible binding sites of E2F4 in GAS5 promoter

Model ID	Model name	Score	Relative score	Start	End	Strand	Predicted site sequence
MA0470.1	E2F4	5.773	0.836	789	799	-1	AGGCAGGAGAA
MA0470.1	E2F4	4.619	0.818	940	950	1	AGGCGTGAGCC
MA0470.1	E2F4	4.206	0.811	1049	1059	-1	AGGAGCGAAAG
MA0470.1	E2F4	6.297	0.845	1072	1082	1	TGGCGGTAACA

4 putative sites were predicted with these settings (80%) in sequence named NC_000001.11:c226408100–226360691

### The expression of GAS5 in cytoplasm could be elevated by rapamycin

**Fig. 6:**
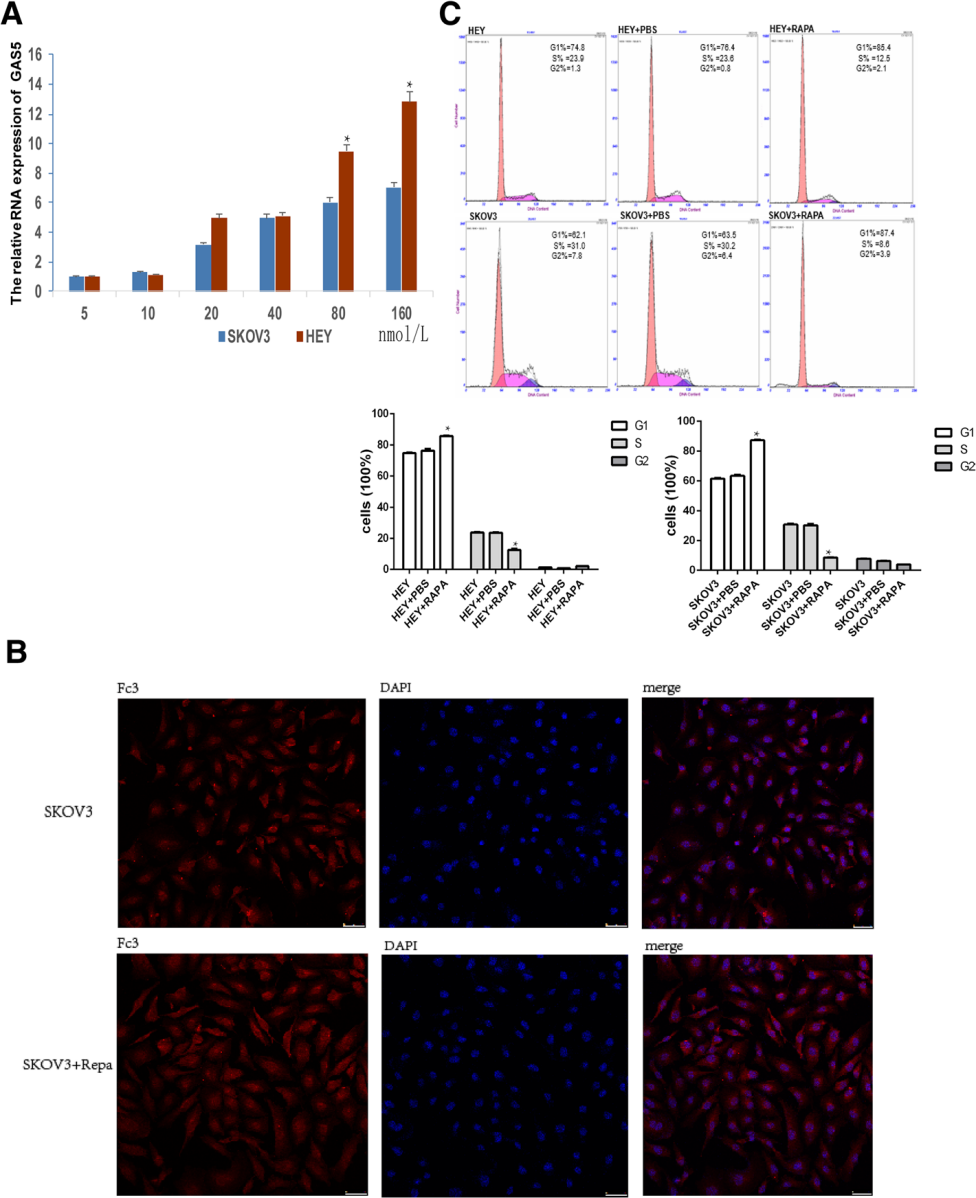
The expression of GAS5 in cytoplasm could be elevated by Rapamycin. **a** The mRNA and protein level of GAS5 were assessed by RT-qPCR in OC cell lines treated by Rapamycin. **b** Cy3-labeled GAS5 and DAPI-labeled U6 probes were obtained from RiboBio. RNA FISH were performed using fluorescent in situ hybridization kit (RiboBio) according to manufacturer’s instructions. **c** Flow cytometry assay were performed to detect the cell cycle impacted by Rapamycin treating in OC cell lines SKOV3 and HEY. *P < 0.05 versus control groups

Since lncRNA-GAS5 is a 5′ TOP (5′-terminal oligonucleotide) gene [11], translational inhibitors such as rapamycin, cycloheximide, and miconazole can increase the accumulation of GAS5 spliced mRNA [25, 26]. Our RT-qPCR assay confirmed that GAS5 mRNA levels increased in a dose-dependent manner after rapamycin treatment (Fig. 6a), and FISH experiments confirmed that rapamycin treatment caused GAS5 to aggregate in SKOV3 cells (Fig. 6b). And as shown by the results of the flow cytometry, rapamycin treatment caused G0/G1 cell cycle arrest as well (Fig.6c). Here rapamycin might be considered as an intervention therapy against ovarian cancer progression by regulating GAS5 expression, and our findings may lay a theoretical foundation for the development of combination therapy of ovarian cancer.

## Discussion

In recent years, research on tumor drug sensitivity-related lncRNA has been receiving increasing attention. Among them, the studies on tumor metastasis-associated lncRNA MALAT1 is relatively mature. Some studies reported that MALAT1 inhibited E-cadherin expression by binding to highly expressed EZH2 at its 3′ end in colorectal cancer, thereby caused tumor cell epithelial-mesenchymal transition (EMT) and oxaliplatin resistance [27]. Meanwhile, MALAT1 caused tumor cell infiltration and drug resistance in glioblastoma multiforme (GBM), combining standard TMZ treatment with targeted nanocomplex carrying siRNA against MALAT1 substantially enhanced the very poor prognosis for GBM patients [28]. And the use of small interfering RNA knockdown MALAT1 in the treatment of enzalutamide (Enz)-resistant prostate cancer has also entered preclinical studies stage [29]. HOTAIR is another lncRNA that has been studied a lot. Teschendorff AE, etc. [30] reported that HOTAIR is highly expressed in ovarian cancer and is associated with poor prognosis and carboplatin resistance. Özeş AR, etc [31] showed that HOTAIR could affects platinum resistance in ovarian cancer by down-regulating NF-κB inhibitor Iκ-Bα, prolonging NF-κB activity, activating its downstream protein interleukin-6 expression to activate DNA damage response pathway (DDR), ultimately maintained tumor cell genome stability, maintain cell survival and avoided apoptosis.

In addition to HOTAIR, more and more lncRNAs affecting ovarian cancer drug sensitivity have been discovered and studied. Wang F, etc [32] found that lncRNA UCA1 affected platinum resistance in ovarian cancer by targeting the SRPK1/PI3K-Akt signaling pathway. Ji An, etc [33] reported that lncRNA NEAT1 affected paclitaxel resistance in ovarian cancer by recruiting miR − 194 to target ZEB1 and then regulating P-gp and GST. However, compared with other tumors, the function and mechanism study of lncRNA in ovarian cancer is relatively weak. In this study, we selected lncRNA-GAS5 to be dramatically down-regulated in ovarian cancer by microarray assay, and then confirmed the low-expression of GAS5 in EOC tissues and OC cell lines. When detected the expression level of GAS5 in OC cell lines we found that the loss of GAS5 in drug-resistant cell lines was much more evidently than in drug-sensitive cells. These findings leaded us to speculate whether GAS5 could affects OC drug sensitivity. Drug sensitivity tests in vitro and in vivo confirmed our conjecture, exogenous overexpression of GAS5 by retroviral plasmids significantly increased the susceptibility of OC to DDP in vivo and in vitro. The results of the flow cytometry and Western-blot showed that overexpression of GAS5 caused G0/G1 cell cycle arrest, apoptosis increase, and affected the expression of cell cycle and apoptosis-related proteins including PARP1.

**Fig. 7:**
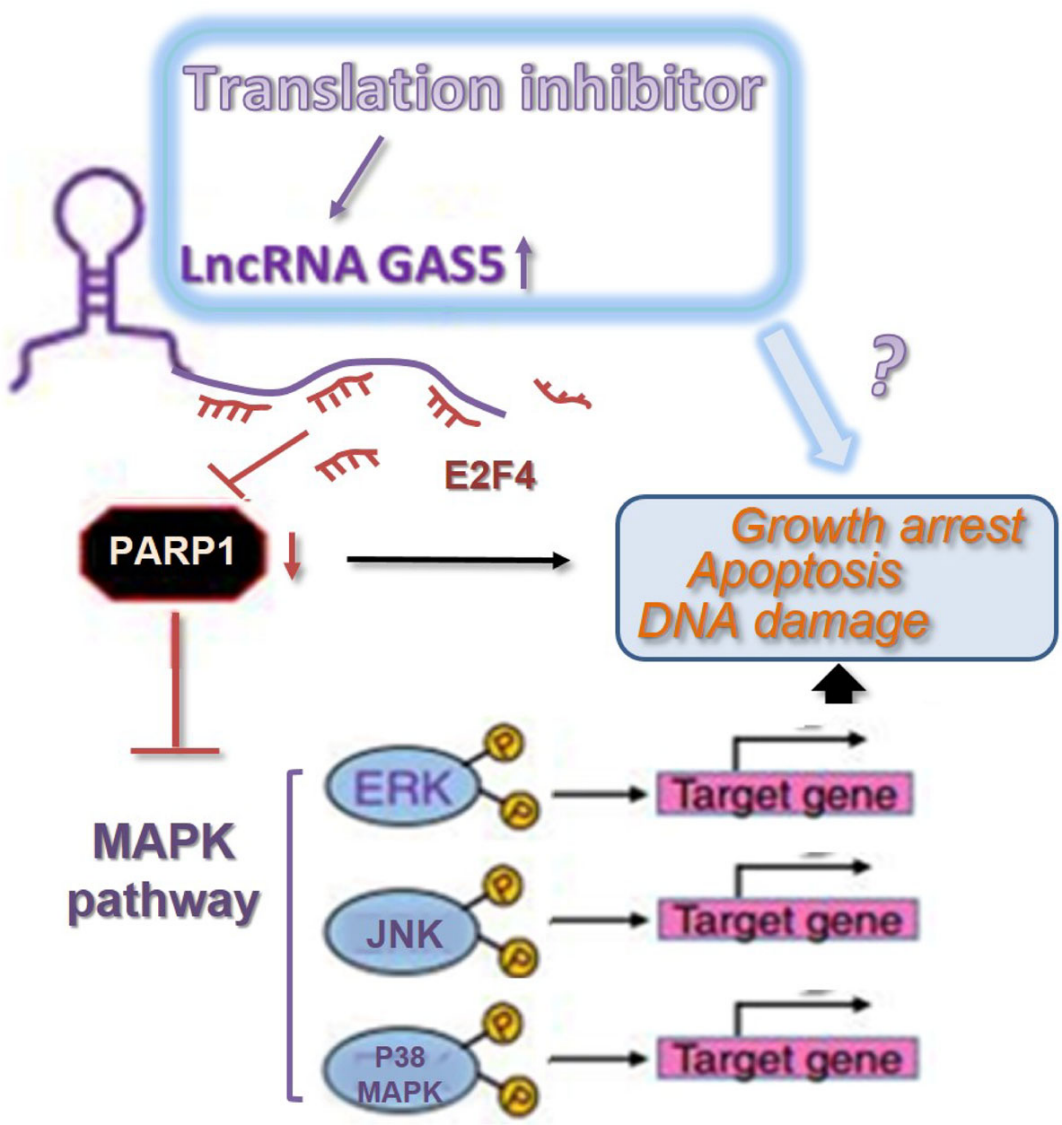
Schematic diagram for GAS5/E2F4/PARP1/MAPK axis in ovarian cancer

Poly-ADP-ribose polymerase 1 (PARP1) is a multitasking enzyme that regulates many intracellular processes, including DNA repair, metabolism, signaling and transcription, by direct interaction with other proteins and DNA [34, 35]. High levels of PARP1 in cancer cells promote cell cycle progression, and may in response to proliferation arrest thereby sensitize cells to agents that challenge redox homeostasis [21]. As for epithelial ovarian cancers, the inhibition of PARP1 even became a promising targeted therapies [36]. PARP1 transcription was reported to be repressed by lncRNAs in lung cancer [37], hepatocellular carcinoma [38], neuroblastoma [39], thyroid cancer [40], multiple myeloma [41], lung morphogenesis [42] and angiogenesis [43]. While in this study, based on our microarray, RT-qPCT and WB data, we found that PARP1 is high-expressed in EOC samples, negatively related with GAS5 expression level, and forced expression of GAS5 markedly down-regulated PARP1 both on protein and mRNA level. The repression of PARP1 transcription by E2F4 complex in human monocytes was reported by Wiśnik E, etc. [22] and Robaszkiewicz A, etc [44] Our luciferase reporter assay showed that E2F4 could decreased promoter activity of PARP1 in OC cells. 13 E2F4-binding sites in PARP1 promoters were predicted by Jaspar matrix models, CHIP assay showed that E2F4’s binding capacity to PARP1 promote was improved evidently with GAS5 overexpression. RNA immunoprecipitation (RIP) assay was performed to demonstrate the E2F4 binding to GAS5. The direct interaction between E2F4 and GAS5 was further verified by GAS5 pull-down assay. While when mutated the binding sites with E2F4, GAS5 lost the regulation capacity on PARP1 expression. Taken together, we concluded that GAS5 inhibited PARP1 transcriptional activity by recruiting transcription factor E2F4 to PARP1 promoter (Fig. 7).

Since lncRNA-GAS5 is a 5′ TOP (5′-terminal oligonucleotide) gene [11], translational inhibitors such as rapamycin, cycloheximide, and miconazole can increase the accumulation of GAS5 spliced mRNA [25, 26]. Here with RT-qPCR and FISH, we confirmed the up-regulation of GAS5 by rapamycin in OC cells. As an intervention therapy against the low-expression of GAS5, rapamycin may take part in the combination therapy of ovarian cancer, but need more investigation.

There are some limitations of our study, first of all, whether rapamycin can affect the apoptosis of ovarian cancer cells by targeting GAS5 expression and how to participate in GAS5 target gene (E2F4 or PARP1) regulation need to be further studied. Secondly, the clinical sample size in this study can be enlarged, especially in the normal group, and the effect of GAS5 expression level on prognosis in different stages of ovarian cancer can be lucubrated. Finally, the function and molecular mechanism of GAS5 can be further verified in GAS5 knockdown OC cell lines.

## Conclusion

In conclusion, we assessed the lncRNA expression profile of EOC, proved that GAS5 worked as a tumor suppressor like gene in OC and might effected DDP sensitivity. We presented the GAS5-E2F4-PARP1-MAPK axis and explored its role in OC progression and drug-sensitivity for the first time. And we proposed that Rapamycin might be used to offset the low expression of GAS5 in ovarian cancer.

## Data Availability

Not applicable.

## Abbreviations

CHIP: Chromatin Immunoprecipitation
DDP: Cisplatin
EOC: Epithelial ovarian cancer
FISH: Fluorescence in situ hybridization
lncRNA: Long non-coding RNA
OC: Ovarian cancer
RIP: RNA immunoprecipitation
RT-qPCT: Quantificational real-time polymerase chain reaction
WB: Western blot
